# Individualized mapping of functional brain networks in older adulthood

**DOI:** 10.1162/IMAG.a.1285

**Published:** 2026-06-29

**Authors:** Colleen Hughes, Anne C. Krendl, Roberto C. French, Shannon L. Risacher, Yu-Chien Wu, Andrew J. Saykin, Richard Betzel

**Affiliations:** Psychological and Brain Sciences Department, Indiana University Bloomington, Bloomington, IN, United States; Department of Internal Medicine, Section on Gerontology and Geriatric Medicine, Wake Forest University School of Medicine, Winston-Salem, NC, United States; Indiana Alzheimer’s Disease Research Center, Indiana University School of Medicine, Indianapolis, IN, United States; Radiology and Imaging Sciences, Indiana University School of Medicine, Indianapolis, IN, United States; Stark Neurosciences Research Institute, Indiana University School of Medicine, Indianapolis, IN, United States; Center for Neuroimaging, Indiana University School of Medicine, Indianapolis, IN, United States; Department of Neuroscience, University of Minnesota, Minneapolis, MN, United States; Masonic Institute for the Developing Brain, University of Minnesota, Minneapolis, MN, United States

**Keywords:** aging, functional magnetic resonance imaging, functional brain networks, resting-state, network segregation, precision functional mapping, resting-state

## Abstract

The functional network architecture of the aging brain undergoes significant systematic and idiosyncratic changes. Emergent individualized network mapping approaches may yield better or more sensitive explanatory insight about age-related neural and behavioral variability, although most applications have focused on young adults. In the current study, we tested the validity and impact of mapping individual-specific topography in two fMRI datasets comprising 112 young (18–35 years) and 176 older adults (60–92 years). Older adults had more idiosyncratic network topography than young adults. Individualized maps from resting-state fMRI improved network homogeneity and fidelity to social cognitive task fMRI activations and exhibited intra-individual stability and inter-individual discriminability over a 2-year interval. Last, traditional group-averaged (*vs*. individualized) network mapping had a moderate-to-large impact on individual-level estimates of network segregation, a widely-studied measure of functional brain aging. Therefore, individualized network mapping captures important heterogeneity in older adulthood and may yield more precise characterization of neurocognitive aging.

## 1 Introduction

A large proportion of the population in many countries now and in the coming decades is entering older adulthood (e.g., 1 in 6 people in the U.S.A. in 2020 (Bureau, n.d.)). For this reason, there is a critical need to understand how aging affects people’s health and daily life to promote longer periods of well-being. Studies of brain aging reveal marked differences across the adult lifespan which, in turn, relate to cognitive decline, the quality of social relationships, and symptomatology in forms of non-normative aging such as dementia (Mwilambwe-Tshilobo et al., 2023; Setton et al., 2023; Wig, 2017; Zhang et al., 2023). A major unifying framework for quantifying brain aging is the organization of brain regions into networks (Sporns, 2013). Brain network architecture revealed by functional connectivity (FC; i.e., inter-regional co-activations during functional magnetic resonance imaging [fMRI]) increases predictive accuracy of individual differences in cognition and behavior compared to anatomical and structural features (Ooi et al., 2022). This advantage of FC may be particularly useful for understanding the greater neural and behavioral inter-individual variability observed in older adulthood (Perez et al., 2025; St-Onge et al., 2023). That said, most studies rely on group-representative networks—whose topography (i.e., spatial extent and boundaries) is determined based on averages of hundreds or even thousands of brains (Yeo et al., 2011)—to calculate and summarize interregional FC (i.e., topology). Emergent research contradicts the idea that group-averaged network maps are a good representation of individuals (Dworetsky et al., 2021; Gordon, Laumann, Adeyemo, et al., 2017; Gordon, Laumann, Gilmore, et al., 2017; Hermosillo et al., 2024; Seitzman et al., 2019). But, few studies have examined this issue among older adults who are broadly characterized by their heterogeneity (Perez et al., 2025) and for whom ignoring this heterogeneity may have pernicious consequences (e.g., poorer sensitivity to cognitive decline).

Capturing individual-specific network topography in older adulthood is vital for a number of reasons (for a review, see Perez et al. (2025)). For instance, areal boundaries based on FC are weaker and more variable in older age, such that even group-averaged network maps made solely on older adults are a poorer fit to a given older individual than a young adult group map would be to a young individual (Han et al., 2018). Age group differences may be exacerbated artificially as a result; an interpretation supported by a recent study showing that group-averaged network maps overestimated the magnitude of differences in FC between individuals with schizophrenia compared to a control group (Levi et al., 2023). Also, a preponderance of studies have identified age-related differences in functional activations when participants actively engage in tasks (Davis et al., 2008; Grady et al., 2006; Spreng & Turner, 2019) and rely on spatial maps of networks for contextualizing and interpreting such findings (and *vice versa*). For example, neural activations are less sensitive to increasing cognitive task difficulty in older (*vs*. young) adults, which was attributed to age differences in network organization (Turner & Spreng, 2015). Yet, recent studies in young and middle-aged adults using individualized network maps find less widespread evidence of overlapping functional neuroanatomy (e.g., for different executive functions) (Assem et al., 2023; DiNicola et al., 2020). That is, functional localization of different cognitive functions within individuals is poorly captured by group-level network maps (Assem et al., 2023). Greater fidelity to individual-specific network borders in older adults may thus reveal why some older adults exhibit more (*vs*. less) cognitive impairment. In sum, studies of neurocognitive aging will gain better or unique explanatory insight about heterogeneity in older adulthood from individualized network mapping, which may improve clinical translation.

Extant longitudinal studies of within-person change during aging typically have dense behavioral and clinical phenotyping but short fMRI acquisitions (e.g., under 20 minutes total fMRI (Villeneuve et al., 2025)). Such datasets thus afford a wealth of information with which to understand the sources and developmental trajectories over time of age-related inter-individual variability in brain and behavior. But short acquisitions limit reliable *de novo* network discovery in individuals (Gordon, Laumann, Gilmore, et al., 2017). To circumvent this issue, another class of approaches leverages observations of the general correspondence of networks across individuals (i.e., *homology*) and the adult lifespan (Han et al., 2018). Specifically, group-based priors are used to initialize network maps that are subsequently refined within individuals (for a review, see (Li et al., 2025)). Template matching is one widely-deployed prior-based approach to generate individualized network maps that has made important basic science and clinical contributions (Gordon, Laumann, Gilmore, et al., 2017; Gratton, Kraus, et al., 2020; Nahas et al., 2025; Seitzman et al., 2019; Siegel et al., 2024). Recent work demonstrated that template matching produced reliable individualized network maps from short fMRI acquisitions in both adults and adolescents (Hermosillo et al., 2024). Few investigations, however, have comprehensively established the validity of individualized mapping when applied to aging populations and how individual variation in network topography differs by age (Perez et al., 2025)—which was the central aim of the current study. Across two datasets, we therefore compared individualized *versus* traditional group-averaged network mapping on several evaluation metrics such as network homogeneity, intra-participant stability, inter-participant discriminability, and alignment of network maps to task activations. We also characterized the extent of individual and age-related variation in network topography, and moreover, how individualization affected a widely-studied FC summary metric linked to higher risk of cognitive impairment in older adulthood (Wig, 2017). By conducting these tests, our objective was to highlight the benefits of individualized network mapping for studying neurocognitive aging and indicate specific challenges to doing so in developmental and clinical populations.

## 2 Methods

In the current work, we analyzed two datasets whose details are summarized in this paragraph. After data quality exclusions, the first dataset comprised 77 older adults (61–92 years) and 112 young adults (18–35 years) recruited as part of a larger study on normative social cognitive aging at Indiana University (IU) Bloomington. Participants underwent 15 minutes of resting-state, 15 minutes of passive movie-watching, and the false belief task (a localizer for social cognition) (Moran et al., 2012) during fMRI on a Siemens 3.0T Prisma Fit MRI scanner. The second dataset comprised 99 cognitively normal adults (60–88 years) recruited and diagnosed at the Indiana Alzheimer’s Disease Research Center (IADRC). The IADRC cohort served to replicate and extend observations in the IU older adult cohort. IADRC participants underwent 10 minutes of resting-state fMRI on a Siemens 3.0T Prisma MRI scanner. For participant demographics, see Table 1. For a summary of the participant workflow across analyses, see Supplementary Figure S1. The individualized network maps were derived from resting-state data only to facilitate comparison between the IU and IADRC samples. All participants provided written informed consent.

**Table 1.** Sample description.

	IU Young adults	IU Older adults	IADRC cohort
	*M (SD) | n*	*M (SD) | n*	*M (SD) | n*
Sample size	112	77	99
Age in years	21.89 (4.17)	72.88 (6.23)	71.33 (5.92)
Age in years (min-max)	18–35	61–92	60–88^a^
Sex (F/M/non-binary)	69 / 40 / 3	52 / 25 / 0	73 / 26 / 0
Race			
White	80	75	68
Black	2	0	30
Asian	20	2	—
Multiracial	10	0	—
Other	—	—	1
MoCA	27.61 (1.90)	26.60 (2.48)	25.72 (2.78)
Education^b^			
High school or less	20	1	11
Some college / no degree	56	12	20
College degree	21	21	54
Advanced degree	15	41	14
% Censored frames			
Rest	8.82 (9.17)	13.74 (10.03)	3.57 (4.43)
Movie run 1^c^	7.13 (8.59)	10.17 (8.74)	—
Movie run 2^c^	6.73 (8.97)	9.74 (8.47)	—
False belief task run 1^c, d^	0.77 (1.79)	2.68 (3.27)	—
False belief task run 2^c, d^	1.23 (2.59)	2.17 (2.91)	—
Residual filtered FD: Rest	0.036 (0.01)	0.043 (0.01)	0.031 (0.012)
Median # of contiguous low motion frames: Rest	80.32 (129.58)	24.34 (41.03)	199.68 (204.81)

*Note.* Montreal Cognitive Assessment (MoCA) (Nasreddine et al., 2005).

a Exact visit age in years was unavailable for 2 participants.

b Education level was unavailable for two IU older adults.

c Only some participants qualified for analyses using these scans – see Supplementary Figure S1 and Section 2.

d While rest and movie-watching were censored using a 0.1 mm filtered framewise displacement threshold, the false belief task was censored using a different threshold of 0.9 mm framewise displacement appropriate for GLM estimation of task fMRI contrasts (Siegel et al., 2014). Residual filtered framewise displacement refers to the mean of low motion frames after censoring. The motion metrics were highly correlated (Supplementary Table S4), and all had significant group differences between IU young adults and IU older adults, *d*s > 0.54, *p*s < 0.001.

### 2.1 IU Bloomington young and older adults: Participant information and image acquisition

#### 2.1.1 Participants

At Indiana University (IU) Bloomington, young and older adult participants were recruited as part of a larger study on social cognitive aging. Older adult participants were pre-screened for cognitive impairment upon recruitment via a telephone-based, well-validated six-item screener (Callahan et al., 2002). A subset of participants without contraindications (e.g., claustrophobia) underwent neuroimaging. Data collection was approved by the Indiana University Institutional Review Board (#11801), including written informed consent from each participant. For the current analyses, participants were included if they underwent neuroimaging (108 older adults, 123 young adults). Of that subset, participants were excluded if they did not complete the resting-state scan or if it had poor image quality (e.g., due to high motion – see section 2.3.1.1; 31 older, 11 young). The analyzed sample comprised 112 young adults (18–35 years) and 77 older adults (61–92 years); see Table 1 for sample demographics. Older adults (range: 19–30) had lower Montreal Cognitive Assessment scores (Nasreddine et al., 2005) compared to young adults (range: 22–30), *t*(182) = 3.17, *p* = 0.002, *d* = 0.47, 95% CI [0.16, 0.75].

#### 2.1.2 Image acquisition

Neuroimaging was performed with a 20-channel head/neck coil on a Siemens 3.0T Prisma Fit MRI scanner at the Indiana University Imaging Research Facility in Bloomington, Indiana. During the 15 minutes resting-state scan, the projector was turned off (no fixation was presented) and participants were instructed to stay awake and keep their eyes open; no other instructions were given. The movies—two episodes of the mockumentary-style television show Nathan for You (each approximately 8 minutes long; for more information, see Hughes et al. (2025)) —were next presented sequentially using MATLAB version 2022b through a Dell laptop running Windows 10 and a projector illuminating a screen that was visible to participants through a mirror attached to the head coil. Then, participants completed two runs (each 5 minutes 20 seconds) of the false belief task, a well-established localizer for neural activity related to theory of mind *versus* non-social narrative comprehension (DiNicola et al., 2020; Saxe et al., 2006; Zaitchik, 1990), which was administered using E-Prime version 3; see Cassidy et al. (2021) for detailed procedural information. In brief, participants read short written vignettes that required them to make inferences about another person’s mental state (theory of mind) or objects (non-social control condition) by judging subsequently presented statements about the vignettes as true or false; responses were made in the scanner using a button box. Participants viewed a total of 12 vignettes for each condition in a randomized order (i.e., event-related design) across two runs.

The T1w anatomical scan was acquired with a high-resolution 3-D magnetization prepared rapid gradient echo sequence (MPRAGE; 160 sagittal slices, 1.0 mm^3^ isotropic voxels, TE = 2.7 ms, TR = 1800 ms, flip angle = 9°, no acceleration). fMRI scans were collected using a gradient-echo echo-planar image (EPI) sequence sensitive to blood oxygen level dependent (BOLD) contrast (T2*; 450 volumes; 54 interleaved slices, 2.5 mm^3^ isotropic voxels, TE = 30 ms, TR = 2000 ms, flip angle = 70°, A/P phase encoding direction, multi-band acceleration factor = 2). Prior to all functional imaging, phase-encoding polarity reversed (PEPOLAR) spin-echo EPI image pairs were acquired for distortion correction (TE = 58 ms, TR = 7700 ms, flip angle = 90°; image and voxel dimensions matched the resting-state parameters).

A subset of older adult participants underwent an identical resting-state fMRI protocol at the same site and scanner approximately 2 years later. The movie-watching and false belief task scans were not repeated. The image acquisition parameters for the follow up MPRAGE T1w anatomical were the same as above, with the addition of generalized auto-calibrating partially parallel acquisition (GRAPPA) acceleration factor = 2.

### 2.2 IADRC: Participant information and image acquisition

#### 2.2.1 Participants

IADRC participants were recruited as part of the embedded Indiana Memory and Aging Study (IMAS) and a separate study on social connectedness. Longitudinal clinical, neuropsychological, and MRI data collection took place at Indiana University School of Medicine, Indianapolis. Data collection was approved by the Indiana University Institutional Review Board, including written informed consent from each participant. The analyzed sample comprised 99 older adults (60–88+ years; Montreal Cognitive Assessment scores range: 17–30, 2 not available) who were categorized as cognitively normal by the IADRC Clinical Core team of psychiatrists, neurologists, and neuropsychologists. Only MRI visits that occurred within 1 year of a cognitive testing session were analyzed, and the most recent MRI was used unless it was deemed poor quality (e.g., due to motion—see section 2.3.1.1; Supplementary Fig. S1).

For longitudinal analysis of topography, a subset of 22 participants (58–77 years) was selected for analysis because they had at least two good quality MRI sessions that were 1–3 years apart. If more than 1 pair of scans met these criteria, we used the most recent scan pair. One participant was 58 at their first visit but over 60 at the follow up visit; the second, more recent scan was used for cross-sectional analyses.

#### 2.2.2 Image acquisition

Neuroimaging for the IADRC cohort was performed on a Siemens 3.0T Prisma MRI scanner with a 64-channel head coil at the Indiana University School of Medicine in Indianapolis. During rest, the projector was turned off (no fixation was presented), and participants were instructed to think of nothing and to remain still with eyes closed. The T1w anatomical scan was acquired with a MPRAGE sequence in accordance with the Alzheimer’s Disease Neuroimaging Initiative (http://adni.loni.usc.edu; ADNI2: 220 sagittal slices, 1.1 × 1.1 × 1.2 mm^3^ voxels, GRAPPA acceleration factor of 2; or ADNI3 [n = 25, scans after June 2023]: 1 mm^3^ isotropic voxels). Fluid-attenuated inversion recovery (FLAIR) scans were incorporated into the Freesurfer workflow to refine estimation of the pial surface, when available (n = 12 without). One run of resting-state fMRI lasting 10 minutes and 7 seconds was collected using a gradient-echo EPI sequence sensitive to BOLD contrast (T2*; 500 volumes, 54 interleaved slices, 2.5 mm^3^ isotropic voxels, TE = 29 ms, TR = 1200 ms, flip angle = 65°, A/P phase encoding direction, multi-band acceleration factor = 3). Prior to resting-state fMRI, PEPOLAR single-echo EPI image pairs matching the resting-state acquisition parameters (excepting TE = 49.8 ms, TR = 1560 ms) were acquired for distortion correction.

### 2.3 IU and IADRC: Image preprocessing

Image preprocessing followed the same procedures across the IU and IADRC datasets, except where noted. Initial anatomical and functional image preprocessing was performed using *fMRIPrep* (IU: v23.2.0; IADRC: v24.1.1) (Esteban et al., 2019) (RRID: SCR_016216), which is based on Nipype 1.8.6 (Esteban et al., 2022; Gorgolewski et al., 2011) (RRID: SCR_002502). In the IU cohort, functional scans across rest, movie-watching, and task states were processed similarly (unless otherwise noted) for straightforward comparison. In the IADRC cohort, the only functional scans that were analyzed were resting-state. In brief, functional images were realigned to correct for motion, underwent slice-timing correction, underwent distortion correction based on two echo-planar imaging references, and were realigned to a corresponding T1w image. The BOLD timeseries were resampled onto the left/right-symmetric template “fsLR” using Connectome Workbench (Glasser et al., 2013). Grayordinates files in connectivity informatics technology initiative (CIFTI) format containing 91k samples were also generated with surface data transformed directly to fsLR space and subcortical data transformed to 2 mm resolution MNI152NLin6Asym space. All resamplings were performed with a single interpolation step by composing all the pertinent transformations.

#### 2.3.1 Denoising rest and movie-watching fMRI

The resting-state and movie-watching scans from both datasets underwent subsequent denoising – appropriate for FC analyses and similar to extant individualized network mapping work (Chernicky et al., 2026; Demeter et al., 2025; Dworetsky et al., 2021; Hermosillo et al., 2024; Lynch et al., 2024)—using the eXtensible Connectivity Pipeline-DCAN (XCP-D, v0.11.1) (Ciric et al., 2018; Satterthwaite et al., 2013). Denoising steps included: (1) discarding the first two volumes of each run to account for potential scanner inhomogeneities, (2) nuisance regression of the *fMRIPrep*-calculated confounding timeseries for the three region-wise global signals extracted within the cerebrospinal fluid, white matter, and global masks, six head motion parameters, the temporal derivatives and quadratic terms for those nine terms, linear trend and intercept terms (Parkes et al., 2018; Satterthwaite et al., 2013), (3) temporal band-pass filtering (0.009–0.08 Hz), and (4) censoring high-motion outlier time points using filtered framewise displacement (fFD) greater than 0.1 mm (Gratton, Dworetsky, et al., 2020)—a threshold chosen to conservatively mitigate noise while balancing loss of temporal degrees of freedom in developmental samples who exhibit higher motion (Chernicky et al., 2026; Demeter et al., 2025; Hermosillo et al., 2024). Last, the denoised data were spatially smoothed with a 6 mm full width at half maximum (FWHM; σ = 2.55) geodesic Gaussian kernel using Connectome Workbench (Glasser et al., 2013).

##### 2.3.1.1 Image quality assessment

*fMRIPrep* reports were manually inspected. Two IU participants (1 older and 1 young) failed Freesurfer-based segmentation during *fMRIPrep* and were thus excluded from analysis. Participants were further flagged for exclusion if their resting-state scans were incomplete or had 5 or more minutes (of 15 total minutes) of frames deemed high motion outliers (i.e., exceeding the 0.1 mm filtered framewise displacement threshold for censoring) —10 young and 30 older adults. In the analyzed IU sample, older adults had more censored resting-state frames than young adults, *t*(187) = 3.49, *p* < 0.001, *d* = 0.51, 95% CI [0.23, 0.85]; which is typical in age cohort studies. See Table 1 for descriptive statistics about the percent of censored frames in each group and scan.

In the IADRC cohort, participants were excluded if concurrent visit age and cognitive status (within 1 year of MRI acquisition) were unavailable, their resting-state scans were incomplete, or resting-state scans had 2 or more minutes (of 10 total minutes) of frames deemed high motion (i.e., exceeding the 0.1 mm filtered framewise displacement threshold for censoring). The cutoffs for quantity of censored data differed between the IU and IADRC to account for multiple factors: (1) different total acquisition times, (2) relative proportion of excluded (vs. included) older adult participants due to high motion at a conservative censoring threshold, and (3) findings from a prior study that argue for maximizing data quantity used for network mapping relative to an approximate minimum of 8 minutes of continuously sampled fMRI data (the amount of data used for IADRC network maps) to achieve discriminability (intra-participant reliability higher than inter-participant similarity) (Hermosillo et al., 2024).

### 2.4 IU & IADRC datasets: Detailed individualized mapping procedure

**Fig. 1.**
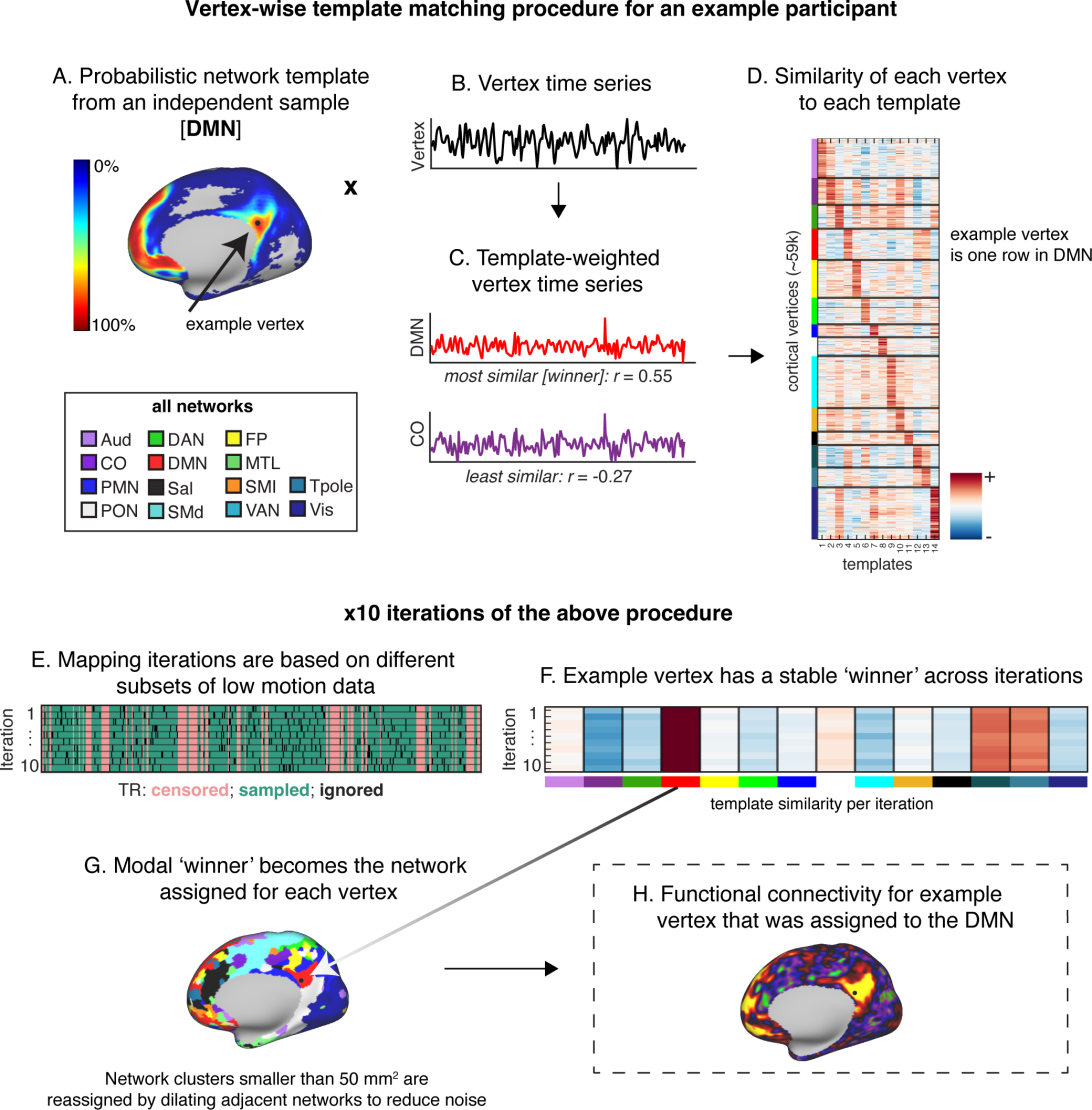
Template matching procedure for individualized mapping of well-characterized functional brain networks. *Note*. Schematic of the vertex-wise template matching procedures described in detail in Section 2. Panel A shows a probabilistic network map from Dworetsky et al. (2021) where values indicate the percent of Human Connectome Project participants (n = 384) for whom each vertex was assigned to the Default Mode Network (DMN). The location of an exemplar vertex, referenced in other panels, is shown. Panel B shows the procedure for comparing the observed vertex-wise activation timeseries to the template-weighted timeseries for each network with examples of the most and least similar templates to that vertex in Panel C. Panel D shows the intermediary output–a correlation matrix–of all vertex-network comparisons to visually describe the winner-take-all network assignment procedure. The y-axis shows vertices sorted by their winner assignment for visual clarity. Panel E shows how temporal subsampling of low motion time points was conducted. Panel F shows the results of 10 iterations of the temporal subsampling and template matching procedures for the exemplar vertex. Panel G shows the right medial surface of the resultant individualized network map using the modal winner across iterations. Panel H shows the functional connectivity of the exemplar vertex also on the right medial surface to show its correspondence to the network map in panel G.

**Fig. 2.**
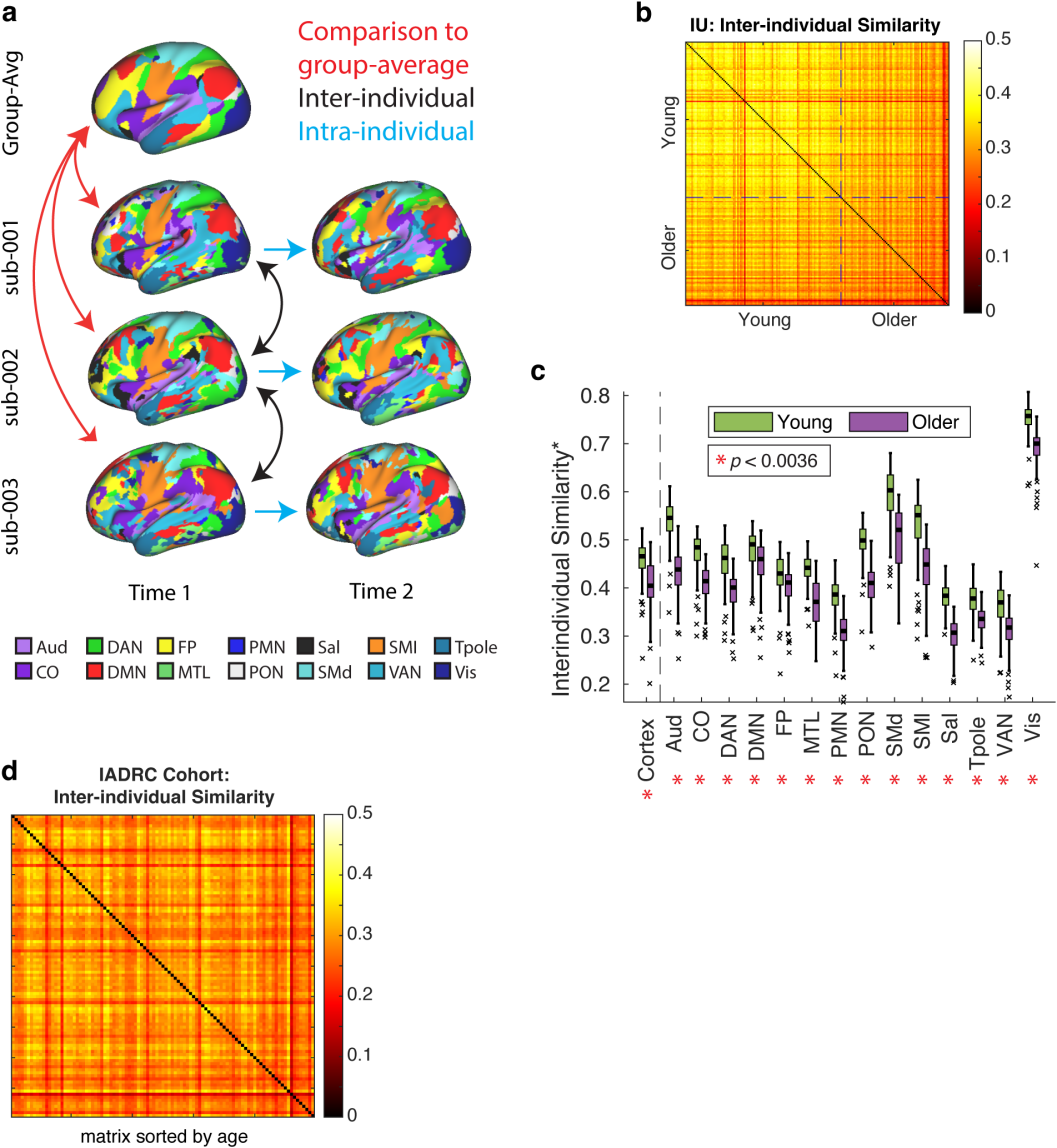
Older adults are more idiosyncratic than young adults. *Note*. (a) Schematic of the analytic approach. We performed three types of comparisons. We compared group-averaged network maps to individualized maps (red arrows); individualized maps from one participant to those of another (black arrows); individualized maps from a single participant at time 1 with maps from the same participant at time 2 (blue arrows). (b) IU cohort (Older: n = 77; Young: n = 112): A participant × participant matrix indicating inter-individual similarity (normalized mutual information) across cortex where rows/columns were sorted by age in years to show how the oldest older adults have the least inter-individual similarity to their peers. On-diagonal elements (i.e., self-comparison) are not applicable. (c) IU cohort: Boxplots indicating age group differences in inter-individual similarity for cortex and, to the right of the dashed line, each individual network (*dice coefficient for binary vectors). Age differences in inter-individual similarity at the Bonferroni-corrected *p*-value of 0.0036 are noted with an asterisk. (d) IADRC cohort (n = 99): Replication of panel (b) that older age related to less inter-individual similarity.

In the current report, we used a variant of the template matching approach to estimate individual-specific network topography of 14 well-characterized functional brain networks (Dworetsky et al., 2021; Gordon, Laumann, Gilmore, et al., 2017; Hermosillo et al., 2024; Lynch et al., 2024): the auditory (Aud), cingulo-opercular (CO), dorsal attention (DAN), default mode (DMN), frontoparietal (FP), medial temporal lobe (MTL), parietal medial (PMN), parieto-occipital (PON), salience (Sal), dorsal somatomotor (SMd), lateral somatomotor (SMl), ventral attention (VAN), temporal pole (Tpole), and visual (Vis) networks. In brief, grayordinates for each participant were assigned a single network label based on the similarity of their activation timeseries to network spatial priors (i.e., templates; for a graphical overview, see Fig. 1). Following Lynch et al. (2024), which included a small subset of older adults, the network templates we used were unthresholded probabilistic spatial templates (similar to Supplementary Fig. S8) derived on an independent sample of 384 Human Connectome Project participants (mean age of 29.3 years) with at least 52 minutes of low motion data (www.midbatlas.io) (Dworetsky et al., 2021). Although this independent sample did not comprise any older adults, probabilistic network templates were chosen because they show good correspondence across a variety of samples and data-driven network assignment methods (Dworetsky et al., 2021; Hermosillo et al., 2024). Template matching was conducted on the cortical surface and in subcortical volume (i.e., grayordinates). Networks were assigned at the level of each vertex (cortical surface) using a winner-take-all approach (Gordon, Laumann, Gilmore, et al., 2017): first, iteratively calculating the similarity of the timeseries of that vertex to the timeseries weighted by the spatial probability of each of the 14 network templates, and second, by assigning the vertex to the network with the greatest similarity (Lynch et al., 2024). Examples of the resulting individualized network maps are shown in Figure 2a and Supplementary Figures S2–S3. Analyses focused on cortical maps.

We combined template matching with a sub-sampling procedure to ensure that network estimates were obtained using equal amounts of low motion data, even though older adults had more in-scanner movement. Specifically, for each participant in the IU cohort, we randomly sampled 10 minutes of the available low motion data (total acquisition duration was 15 minutes) and, given these data, performed template matching to obtain an estimate of network assignments. In the IADRC sample, we sampled 8 minutes of the available low motion data; the total acquisition duration was 10 minutes. We repeated this procedure 10 times, as in Hermosillo et al. (2024), yielding 10 versions of the network maps for each participant. The consensus network label for each grayordinate was its modal assignment across those versions (i.e., the network to which a vertex was the most frequently assigned). Last, network clusters smaller than 50 mm^2^ were reassigned by dilating adjacent network clusters using Connectome Workbench (Glasser et al., 2013), which falls within the range of thresholds used in this literature (Dworetsky et al., 2021; Gordon et al., 2020; Hermosillo et al., 2024).

### 2.5 IU dataset: Theory of mind localizer analysis

The *fMRIPrep* cifti-formatted false belief task outputs were spatially smoothed with a 6 mm FWHM (σ = 2.55 sigma) geodesic Gaussian kernel using Connectome Workbench (Glasser et al., 2013) and then submitted to task general linear model (GLM) analysis at the individual-level. The data were modeled using *FitLins* (v.0.11.0) (Markiewicz et al., 2022), a wrapper for nilearn-based estimation of task GLMs that was developed to take BIDS-style inputs from *fMRIPrep* and analyze them according to a BIDS Stats Model file (Markiewicz et al., 2021) to improve the reproducibility of task fMRI analysis. For each participant and run, we modeled the task as a GLM with four conditions reflecting the trial type (theory of mind, control) × trial component (read story, make inference), as well as covariates of no interest from the *fMRIPrep*-derived confounds file: 6 head motion parameters, non-steady state volumes, and volumes exceeding 0.9 mm framewise displacement (Siegel et al., 2014). For each participant, we localized neural activity specific to theory of mind using the average theory of mind > control *z*-value contrast images across runs (DiNicola et al., 2020; Moran et al., 2012) (Fig. 4a, left). Participants were excluded from localizer analysis if either or both task runs were not acquired (e.g., due to time constraints, 4 young, 2 older), or if either run had 25% or more volumes flagged as a high motion outlier (2 young, 4 older).

## 3 Results

### 3.1 Older adults have more idiosyncratic network maps than young adults

First, we tested the central premise of the current work that network topography of older adults would be more different from the group-averaged map compared to young adults. Because most group-averaged network maps in the literature were not created by sampling older adults (e.g., (Yeo et al., 2011); c.f. (Chernicky et al., 2026; Han et al., 2018)), which could introduce bias for age group comparison, we created group-averaged maps from subsets of participants in the current datasets. Specifically, for the IU sample, we constructed the group-averaged network mapping by applying an analogous template matching procedure to group-averaged FC from a subset of the 50 lowest motion older adults and 50 motion-equated young adults (Supplementary Fig. S4). We also created an IADRC-specific group-averaged network map from a subset of the 50 lowest motion IADRC participants (Supplementary Fig. S5).

For each participant in the IU cohort, we quantified the normalized mutual information (NMI; (Meilă, 2007)) – a metric between 0–1 where higher values indicate more similar network assignments on a vertex-to-vertex level—between each individual’s map and the group-averaged network map. As hypothesized, in the IU cohort, older adults (*M* = 0.41, *SD* = 0.05) were less like the group-averaged map than young adults (*M* = 0.46, *SD* = 0.04), *t*(187) = 7.61, *p* < 0.001, *d* = 1.12, 95% CI [0.74, 1.45]. However, because group-averaging induces a smoother (i.e., spatially blurred) and more contiguous network map, we also compared how similar each participant’s network map was to every other participant. Older adults (*M* = 0.28, *SD* = 0.03) were more dissimilar to their peers than young adults (*M* = 0.35, *SD* = 0.03), *t*(187) = 16.83, *p* < 0.001, *d* = 2.48, 95% CI [1.78, 3.02]. Some caution regarding these age differences in peer-to-peer comparisons is warranted. Although young and older adults in the IU sample were recruited from the same community, its nature as a mid-size college town could mean that young adults were more like each other than what may be observed among young adult samples from other communities.

For this reason, we also explored the effect of age within each age cohort. Lower inter-individual topographical similarity related to higher head motion in the IU older adults, *r*(110) = -0.32, *p* = 0.005, 95% CI [-0.56, -0.16]; but not IU young adults, *r*(110) = -0.17, *p* = 0.08, 95% CI [-0.40, 0.03]; or IADRC older adults, *r*(97) = 0.02, *p* = 0.82, 95% CI [-0.13, 0.18]. Therefore, we calculated the partial correlations between age and inter-individual topographical similarity controlling for head motion (i.e., the percent of censored frames) in all groups. Notably, lower chronological age related to higher inter-individual similarity among IU older adults, controlling for head motion, *r*(75) = -0.52, *p* = 0.005, 95% CI [-0.65, -0.35] (Fig. 2b). In the IADRC replication cohort, lower chronological age also related to greater inter-individual similarity, *r*(97) = -0.26, *p* = 0.01, 95% CI [-0.46, -0.03] (Fig. 2d). In contrast, IU young adults had less variability in chronological age which may explain why it was unrelated to their inter-individual similarity, *r*(110) = -0.15, *p* = 0.11 95% CI [-0.37, 0.01].

Both global and network-specific changes in FC are observed in older adulthood (Betzel et al., 2014; Chan et al., 2014; Setton et al., 2023). As such, we next compared inter-individual similarity of specific networks to test the possibility that some, but not all, networks exhibit greater topographical variation in older adults. Each participant’s individualized network map was a vector of values 1–14 indicating the network assignments of each of 59,412 cortical vertices. For each network, we binarized the vector (1 = target network, 0 = any other network) and compared the binarized vectors using the dice coefficient (Dworetsky et al., 2021). We found that all networks exhibited reduced inter-individual similarity in older *versus* young adults, *t*s > 4.26, *p*s < 0.001 (Fig. 2c). Taken together, these results suggest that older adults had greater topographical heterogeneity than young adults, which would otherwise not be captured in a group-averaged network map.

### 3.2 Individualized network maps increase network homogeneity

**Fig. 3.**
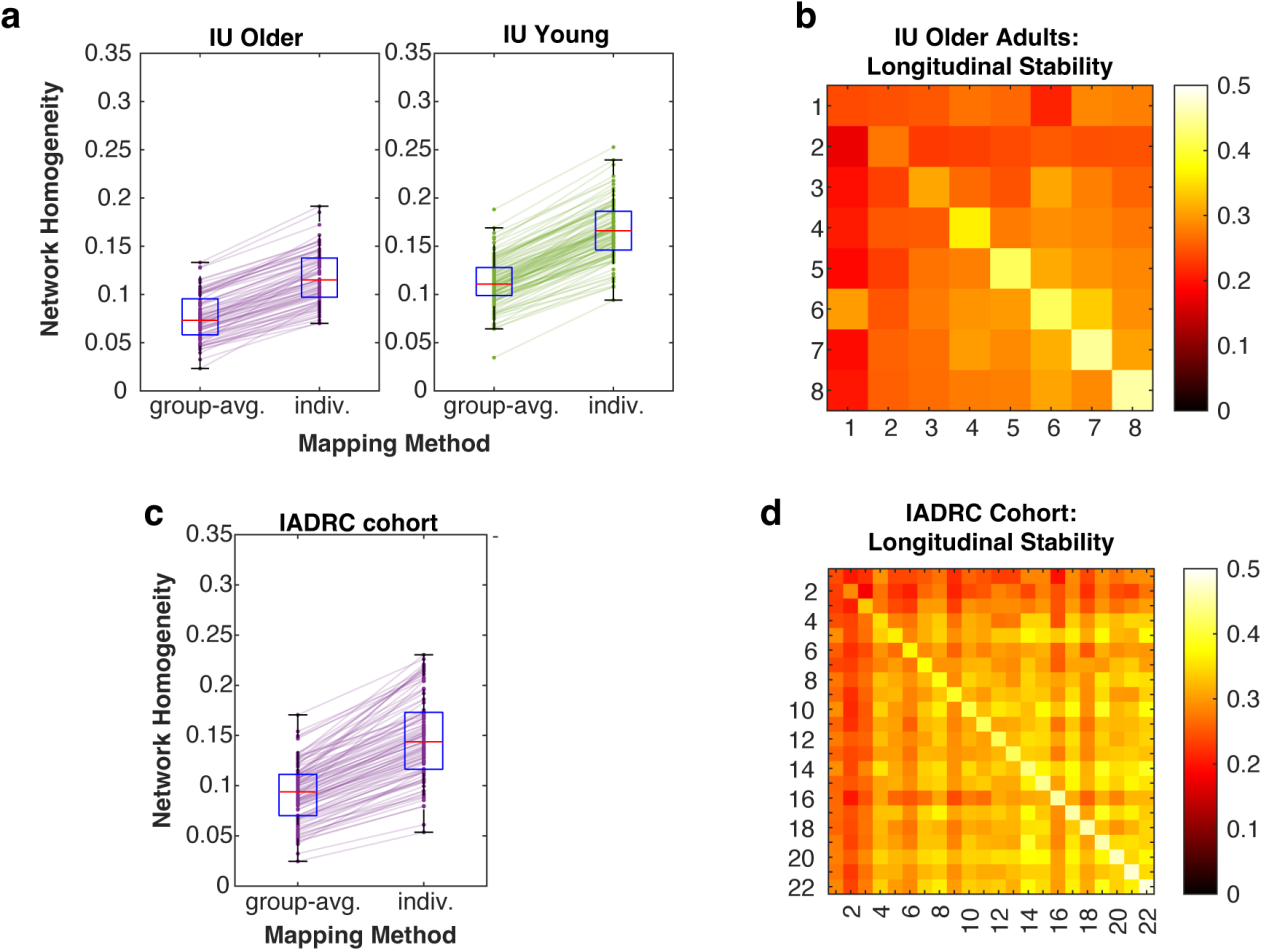
Individualized network maps increased network homogeneity, and intra-individual stability over a 2-year interval was higher than inter-individual similarity. *Note*. Network homogeneity—the strength of the correlations between vertices within networks – was higher for individualized *versus* group-averaged network mapping in (a) IU older and young adults and (c) the IADRC cohort. Individualized network maps were moderately stable and discriminable (i.e., more like self than anyone else) over a 2-year interval in a subset of IU cohort older adults (n = 8; b) and IADRC cohort older adults (n = 22; d) who underwent repeated imaging. The diagonal indicates intra-individual stability over time. The upper (time 1) and lower (time 2) triangles indicate inter-individual similarity at each time point.

In the previous section, we showed that older adults had more idiosyncratic network topography than young adults. It remained unclear, however, if this finding reflected meaningful variation. To establish the validity of individualized network maps, we examined several metrics (for a review, see Perez et al. (2025), Li et al. (2025)) First, we confirmed that vertices with similar FC profiles were appropriately grouped together by the template matching procedure for individualization. To do so, we computed a measure of network homogeneity by taking the mean product moment correlation between every pair of vertices within the same network (e.g., DMN-DMN but not DMN-VIS), following past work (Gordon et al., 2020; Hermosillo et al., 2024). We compared homogeneity values from the vertex-by-vertex dense FC matrix (59,412 × 59,412 cortical vertices) that was divided into networks based on both an individual’s individualized map as well as the group-averaged map. We did so by comparing the observed *t*-value to a permuted null distribution created by randomizing the correspondence of mapping method labels to values across 10,000 iterations. Model *p*-values were considered statistically significant if they fell below a Bonferroni-corrected threshold of *p* = 0.0036 (α = 0.05 divided by 14). The individualized maps had greater homogeneity than the group-averaged maps in both age groups across cortex and in most, but not all (i.e., posterior medial network), networks (Fig. 3a, Supplementary Fig. S6a, Supplementary Table S1). This finding, including non-significance of the posterior medial network, was replicated in the IADRC cohort (Fig. 3c, Supplementary Fig. S6b, Supplementary Table S2).

In young and older adults from the IU sample, we also calculated cross-task network homogeneity from resting-state maps applied to dense FC from independent movie-watching fMRI scans. Supporting intra-participant stability, we again found higher cortical network homogeneity from the individualized versus group-averaged maps across age groups (Supplementary Fig. S7, Supplementary Table S3). The overall effect was smaller than within-state homogeneity and network-level effects varied, which may be unsurprising because older adults have more dissimilar rest-task FC than young adults (Hughes et al., 2025). Global and network-specific improvements in network homogeneity confirmed that the template matching approach optimized cohesiveness within distinct networks, while preserving inter-participant network homology (i.e., all networks were represented in all participants, which aids inter-participant comparison). While homogenous functional networks are often the goal of network discovery methods, we next conducted tests that specifically validated the stability and uniqueness of individual-specific resting-state topography.

### 3.3 Intra-individual stability is higher than inter-participant similarity across a 2-year interval

Next, we determined whether individualized network maps were stable within individuals and discriminated between individuals over time (i.e., individuals were more like their own network maps than anyone else). Although FC weights (i.e., topology) using group-averaged network definition have been shown to be stable and discriminable within and across testing sessions spanning months to years (Chernicky et al., 2026; Horien et al., 2019; St-Onge et al., 2023), few studies have examined this question for individual network topography in older adults either cross-sectionally or longitudinally. To address this gap, we leveraged a subset of older adult participants in both the IU and IADRC cohorts who underwent imaging two or more times approximately 2 years apart. We chose this interval because it is common to longitudinal studies of aging (e.g., (Villeneuve et al., 2025)). In the IU cohort, 8 older adults (4 male, 4 female, time 1: ages 68–81, *M* = 74.48, *SD* = 4.24) were recruited 2 years (698–768 days) after their first participation to undergo an identical resting-state fMRI protocol. Individualized network maps were created following the same procedures described above. The maps within individuals over time (intra-individual stability) and between individuals (inter-individual similarity) were compared using the above-described NMI metric. Specifically, inter-individual similarity was calculated between each pair of participants at each of the two timepoints. Then, we took the average per pair across timepoints. Finally, we took the average across participants in the time-averaged inter-individual matrix, resulting in a 1:1 ratio of intra-individual to inter-individual observations. Statistical significance was determined by permuting values associated with intra-individual and mean inter-individual comparisons. To aid interpretation of NMI, we reported the percent of shared versus non-shared network assignments for the same pairwise comparisons.

In the IU older adult longitudinal cohort (n = 8), participants were more like themselves over time (NMI: *M* = 0.37, *SD* = 0.08; Same-network vertices: *M* = 52.70%, *SD* = 6.92%) than like others (NMI: *M* = 0.26, *SD* = 0.02; Same-network vertices: *M* = 42.41%, *SD* = 1.63%), *t*(7) = 3.42, *p* = 0.005, *d* = 1.62, 95% CI [0.54, 3.27] (Fig. 3b). Directly, 7 of the 8 participants in the longitudinal were more like themselves than anyone else at either time point (Fig. 3b). In the IADRC longitudinal cohort (n = 22, 6 male, 16 female, time 2: ages 58–77, *M* = 68.76, *SD* = 4.73) whose imaging sessions were approximately 2 years apart (379–994 days, *M* = 610.55, *SD* = 186.50), we replicated higher intra-individual stability (NMI: *M* = 0.40, *SD* = 0.06; Same-network vertices: *M* = 56.53%, *SD* = 5.92%) than inter-individual similarity (NMI: *M* = 0.30, *SD* = 0.03; Same-network vertices: *M* = 45.70%, *SD* = 2.21%) over time, *t*(21) = 7.13, *p* < 0.001, *d* = 2.11, 95% CI [1.02, 3.12] (Fig. 3d). Directly, 19 of the 22 participants in the IADRC longitudinal were more like themselves than anyone else at either time point (Fig. 3d).

These findings demonstrate that individualized mapping is a feasible strategy to deploy in longitudinal studies of within-person change. See the Supplementary Material for similar analyses, conducted within a single testing session by pooling rest and passive movie-watching fMRI data (Du et al., 2025; Hermosillo et al., 2024; Wang et al., 2015), that compare young *versus* older adults.

### 3.4 Individualized network mapping explains more variance in theory of mind task activation patterns compared to group-averaged network mapping

Findings of overlapping functional neuroanatomy from task fMRI studies inform theoretical models across domains (Assem et al., 2023; Lighthall, 2020; Spreng et al., 2009). For example, activity in default network regions is elicited by tasks that require participants to think about themselves in the past/future and about other people, which can be taken as evidence that personal experiences facilitate understanding of other people’s thoughts, feelings, and behavior (i.e., theory of mind (Hughes et al., 2024; Spreng et al., 2009)). But, group-averaged mapping methods may overestimate the shared neural basis of these forms of social cognition (DiNicola et al., 2020). For this reason, we next tested the alignment between resting-state network boundaries and task activations to validate individualized network mappings (Chong et al., 2017; Du et al., 2025; Li et al., 2025). Specifically, IU young and older adults additionally underwent fMRI while engaging in the well-established false belief task (Cassidy et al., 2021; Saxe et al., 2006; Zaitchik, 1990) for localizing neural activations associated with theory of mind. Theory of mind exhibits robust age-related behavioral impairment (Grainger et al., 2023) and neural differences in activations and FC (Hughes et al., 2019, 2020, 2025), making it well-suited to the current investigation.

**Fig. 4.**
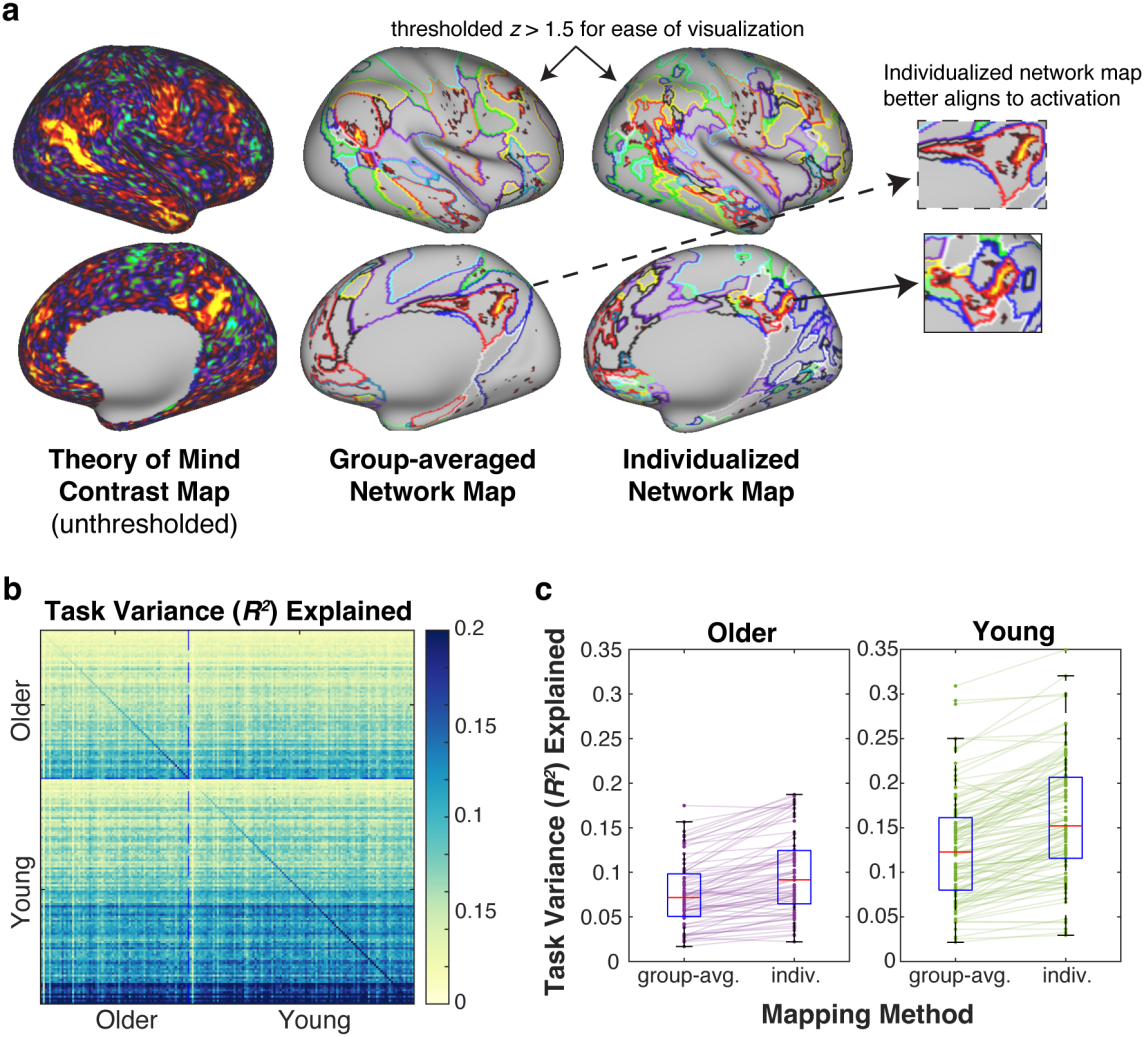
Individualized network mapping improves network-function alignment with theory of mind task fMRI contrast activations in both young and older adults. *Note*. To examine network-function alignment, we first calculated the GLM *z*-contrast [theory of mind condition > control condition] for each participant (a, left). Network-function alignment was operationalized as the variance in the task activation map that was explained by the individualized map (a, right; b, on-diagonal). We compared the observed *R^2^* for each participant to two null models: variance explained by other participants’ individualized maps (b, off-diagonal) and the group-averaged network map (a, middle; c). Under both null models, participants’ own individualized networks explained more task activation variance. The example contrast map and network maps visualized in panel (a) are available in CIFTI format for further inspection (see the Data and Code Availability section).

To measure network-function alignment for each participant, we followed an established spatial ANOVA approach (Gordon et al., 2020) in which activation values for the theory of mind condition > control condition (Fig. 4a) *z*-contrast were predicted by resting-state network assignment using a one-way ANOVA. Variance explained (*R^2^*) was the outcome of interest, where higher values indicated better network-function alignment. We compared the observed *R^2^* values to two null models: other participants’ individualized network maps (Gordon et al., 2020) (Fig. 4b) and the group-averaged network map (Fig. 4c), each applied to a given participant’s contrast map. In both cases, we observed that participant’s own individualized network maps explained more task variance in both older adults, *t*s > 3.37, *p*s ≤ 0.001 and young adults, *t*s > 3.95, *p*s < 0.001. The observed greater variance explained in older adults is noteworthy because older adults tend to have weaker theory of mind activations in regions such as medial prefrontal cortex, precuneus, and temporal poles (Cassidy et al., 2021; Moran et al., 2012) which could have, but did not, limit the greater precision afforded by individualized network mapping. Because only one psychological domain was tested here, leveraging multi-domain task fMRI datasets (Bookheimer et al., 2019) could provide further evidence of the robustness of the current approach. Gaining individual-level specificity to functional neuroanatomy through individualized mapping may thus promote more precise characterization of behavioral and neural deficits occurring in some, but not all, older adults.

### 3.5 Network topography

Having validated the homogeneity, stability, discriminability, and external validity to task activity of individualized network maps in older adults, we next sought to describe age-related topographical similarities and differences that would be otherwise unobservable using group-averaged network maps. We examined three complementary features of age-related network topography: (i) probabilistic similarity, (ii) spatial consensus, and (iii) network size and displacement.

#### 3.5.1 Probabilistic network topography is similar across age groups

Prior reports indicated that some areas of cortex have highly consistent network assignments within and across samples (Dworetsky et al., 2021; Hermosillo et al., 2024). For comparison to this literature, we calculated the probability of each network in each group (i.e., the fraction of participants to which each vertex was assigned to a given network). Qualitatively, the probabilistic maps were similar across age groups (Supplementary Fig. S9). To quantify these observations, we calculated the similarity (i.e., product-moment correlation across all vertices) of the unthresholded probabilistic maps between young and older adults. Indeed, the probabilistic maps between young and older adults were highly correlated across networks (*r*s = 0.96 – 0.99). We observed, however, that older (*vs*. young) adults tended to have less consensus with their peers in both peripheral and some, but not all, central areas of certain networks (e.g., the dorsal anterior cingulate area of the cingulo-opercular network [CO]).

**Fig. 5.**
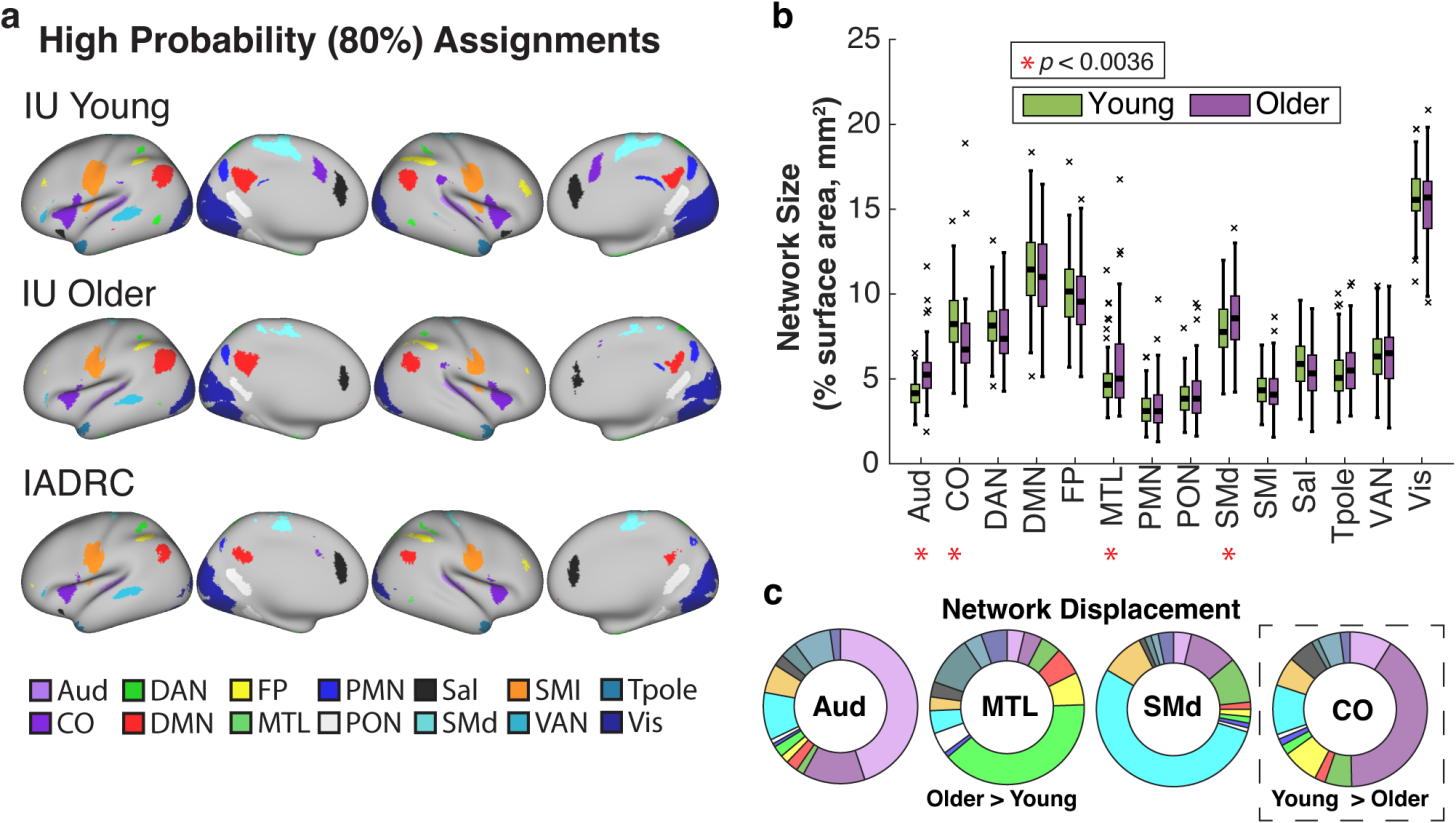
Individualized network topography. *Note*. (a) High probability network assignments in each group using an 80% probability threshold (see also Supplementary Fig. S10, which uses a 60% threshold). (b) Some networks exhibited significant age differences (IU: young *vs*. older) in surface area, noted with an asterisk if below the Bonferroni-corrected *p*-value of 0.0036. (c) For those networks, the donut plots indicate the average percent of vertices where network assignments agreed between groups or not, and if not, which of the other networks was commonly assigned to the same vertex.

It is common to compare individuals in terms of FC and contextualize those results using network labels. However, these network-level summaries may be misleading because many grayordinates have inconsistent network assignments across individuals. As an alternative, recent work has suggested that these types of analyses may benefit by comparing individuals using only those grayordinates with a high level of consensus in their network assignments, leading to improved reliability of brain-behavior correlations (Hermosillo et al., 2024). This prompts the next question that we addressed: are high consensus areas similar between young and older adults? We identified highly consensual network assignments in each IU age group and the IADRC cohort by taking the modal network assignment at each vertex, calculating the probability of that modal assignment, and thresholding to retain vertices with 80% or greater consensus (Fig. 5a). We observed that high consensus areas were broadly similar in location across groups, but the spatial extents (i.e., size) at the 80% threshold tended to be smaller in older adults. This observation was supported by binarizing the thresholded maps to vertices labeled as high consensus or not and comparing their spatial similarity between young and older adults. Young and older adults had lower similarity at the 80% threshold for high consensus regions (dice coefficient = 0.77) than any comparison between 10,000 permutations of random groups, *p* < 0.001. For results at other thresholds, see Supplementary Figures S10–S11. The finding that older adults had less consensus than young adults is consistent with the previously reported finding that their individualized network maps were more idiosyncratic.

#### 3.5.2 Some, but not all, networks differ in size in older versus young adults

Based on recent work in individuals with and without depression (Lynch et al., 2024), another feature of network topography that could differ by age and is uniquely afforded by individualized, but not group-averaged, mapping is network size. Specifically, we calculated the percent of surface area in mm^2^ of vertices assigned to each network in each person (Lynch et al., 2024). Using a Bonferroni-corrected *p*-value threshold of *p* = 0.0036, we found that the following networks were expanded in older *versus* young adults: auditory network, *t*(187) = 6.55, *p* < 0.001; medial temporal lobe network, *t*(187) = 3.14, *p* = 0.001; and dorsal somatomotor network, *t*(187) = 2.94, *p* = 0.0036 (Fig. 5b). Conversely, the cingulo-opercular network was expanded in young *versus* older adults, *t*(187) = 4.60, *p* < 0.001 (Fig. 5b). All other networks did not show a significant difference in size by age group, *t*s < 1.84, *p*s > 0.16. Because extant work varies in reporting network size as a function of the percent of surface area or percent of vertices, we calculated both though they were highly correlated across age groups, *r_mean_* = 0.92, *SD* = 0.03. We also conducted a robustness analysis on the residuals of network surface area after regressing out a participant-level measure of topological complexity – used to quantify surface registration quality (Moqadam et al., 2024; Rosen et al., 2018) – that was higher in older *versus* young adults, *t*(187) = 3.72, *p* < 0.001. The direction and magnitude of age differences in network size residuals were similar, Aud: *t*(187) = 5.88, *p* < 0.001; CO: *t*(187) = 4.02, *p* < 0.001; but the age differences in the medial temporal lobe network, *t*(187) = 2.43, *p* = 0.012, and dorsal somatomotor network, *t*(187) = 2.36, *p* = 0.009, were no longer significant at the Bonferroni-corrected threshold.

To further characterize these age differences in network size, we conducted an exploratory analysis in which we examined which networks were ‘displaced’ when older adults had expanded auditory, dorsal somatomotor, and medial temporal lobe networks compared to young adults and, conversely, when young adults had an expanded cingulo-opercular network compared to older adults. To do so, we compared network assignments for each vertex between each pair of individuals. Of the vertices where the assignments disagreed, we calculated the percent of vertices attributed to the displaced network. Then, to characterize group-level findings, we averaged the displacement matrix (networks × networks) for older adults compared to young adults and vice versa. This approach mirrors past reports, except it compares individuals to each other rather than to a group-averaged network mapping (Lynch et al., 2024; Nahas et al., 2025). Qualitatively, we observed that older adults’ larger auditory network may “trade-off” (exchange border vertices) with the spatially adjacent cingulo-opercular network, which was expanded in young adults (Fig. 5c). Future investigations should consider the physiological, neurobiological, or behavioral antecedents and correlates of these differences (e.g., vascular function (Farahani et al., 2025), precision estimates of structural brain aging (Elliott et al., 2026), or structure-function correspondence, (Han et al., 2018; Rajesh et al., 2024)), as well as potential confounds (e.g., inter-regional temporal signal-to-noise ratio is particularly low in the medial temporal lobe network, Supplementary Fig. S12) when interpreting findings about network size (for more detail, see Section 4 (Dworetsky et al., 2024; Seitzman et al., 2019)).

### 3.6 Group-averaged network mapping overestimates age group differences in network segregation compared to individualized network mapping

Topography and topology are related, raising the possibility that individualized topography leads to different estimates of age differences in topology. As such, we next tested how the effect of individualized *versus* group network topography impacted FC analyses. Specifically, we tested the impact of mapping method on estimates of network segregation, a ratio of within-network FC (homogeneity) to between-network FC (distinctiveness) (Chan et al., 2014). We focused on this FC metric because less network segregation relates to: (1) older age in both cross-sectional (Betzel et al., 2014; Chan et al., 2014) and longitudinal (Chan et al., 2021) studies, (2) less neural activation selectivity (Chan et al., 2017) and less efficient network reconfiguration (Hughes et al., 2025) when participants engage in cognition (Ewers et al., 2021), (3) cognitive deficit and reserve (Chan et al., 2014, 2018, 2021), (4) greater dementia severity (Zhang et al., 2023), and (5) greater tauopathy in Alzheimer’s disease (Steward et al., 2023). Altogether, these findings highlight that network segregation is a robust indicator of neurocognitive aging that captures important heterogeneity among older adults.

**Fig. 6.**
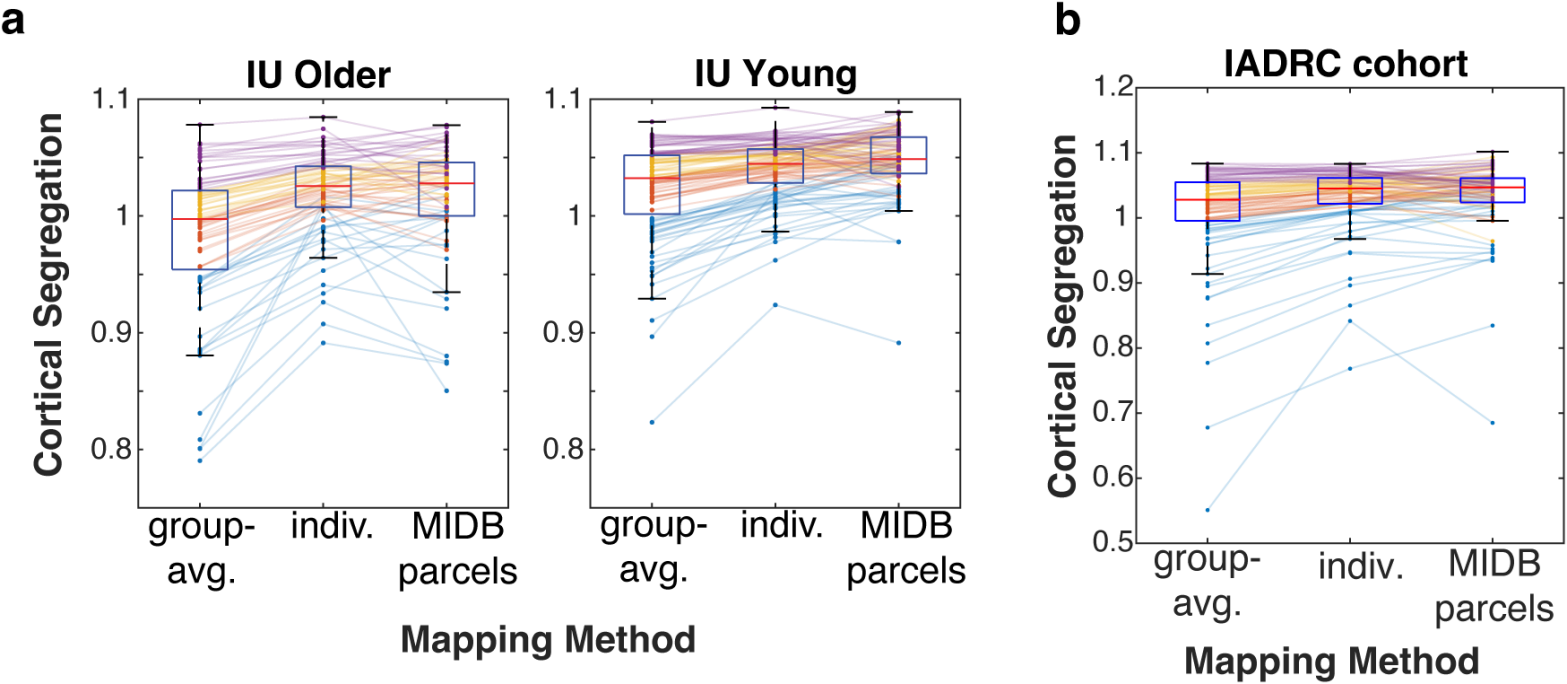
Cortical segregation values by cohort and mapping method (group-averaged network mapping, individualized network mapping, MIDB probabilistic parcellation). *Note*. For the group-averaged (“group-avg.”) and individualized (“indiv.”) mapping methods, segregation was calculated on the dense FC matrix (59,412 cortical vertices). For the MIDB parcellation, segregation was calculated on the 80-parcel FC matrix. Lines represent individual participants and are colored by quartile of segregation values using the group-averaged map. (a) IU older and young adult cohorts. (b) IADRC cohort.

First, we observed that cortical segregation was greater when using individualized *versus* group-averaged network maps in both the IU, *t*(188) = 5.34, *p* < 0.001 (Fig. 6a), and IADRC (Fig. 6b), *t*(97) = 2.47 *p* = 0.012, cohorts. This finding reflects that individualized mapping increases both network homogeneity (stronger within-network FC) and network distinctiveness (weaker between-network FC). Also, we replicated past observations that young adults had higher network segregation than older adults, using both the group-averaged, *t*(187) = 5.29, *p* < 0.001, *d* = 0.78, 95% CI [0.49, 1.09]; and individualized, *t*(187) = 4.61, *p* < 0.001, *d* = 0.68, 95% CI [0.37, 0.96] mapping. We then tested the possibility, consistent with our hypothesis and prior work (e.g., Levi et al. (2023)), that group-averaged (*vs*. individualized) network maps overestimate the magnitude of cohort differences. We tested this possibility with a linear model that included age group, mapping method, and their interaction. We extracted the observed *t*-value for the interaction term. Then, we randomly permuted the mapping method labels to create a null distribution of magnitudes of age differences in segregation due to chance. We found that the group-averaged network mapping method yielded a small but significantly greater magnitude of age group differences in segregation compared to individualized network mapping, *t*(1, 378) = 2.20, *p* = 0.043, η_p_^2^ = 0.013, 95% CI [0.000, 0.048]. Speaking to how segregation might be used to estimate individual risk, we also tested whether older adults had a greater discrepancy in segregation values between mapping methods than young adults. To do so, we created a participant-level difference score in segregation (individualized minus group-averaged), then compared the observed *t*-value between age groups to a null distribution from permuted age group labels. Older adults showed a greater discrepancy between mapping methods than young adults, *t*(187) = 4.90, *p* < 0.001, *d* = 0.72, 95% CI [0.41, 0.98]; Figure 6 highlights that the individuals in the lowest quartile for group-averaged segregation values had the most discrepancy between mapping methods.

Last, we examined how areal parcellation that leverages, rather than dilutes, individual variation in topography—by excluding vertices whose network assignments are less consistent across individuals—affects network segregation. For this comparison, we used Masonic Institute for the Developing Brain (MIDB; www.midbatlas.io) probabilistic parcels to capture only the regions of cortex whose network assignments are highly consensual across individuals and samples (analogous to Fig. 5a) (Hermosillo et al., 2024). Using these 80 regions (14 networks, 2–14 parcels/network), we constructed parcellated FC matrices (80 × 80) for each participant upon which network segregation was calculated. We replicated that young adults had higher network segregation than older adults using the probabilistic parcels, *t*(187) = 5.66, *p* < 0.001, *d* = 0.83, 95% CI [0.50, 1.11]. Unlike the comparison between the group-averaged and individualized methods, there was no evidence that probabilistic mapping affected the magnitude of cohort comparison compared to individualized mapping, *t*(1, 378) = 1.37, *p* = 0.21, η_p_^2^ = 0.005, 95% CI [0.000, 0.031]. Nonetheless, older adults again showed a greater discrepancy between mapping methods (individualized *vs*. probabilistic parcels) compared to young adults, *t*(187) = 2.82, *p* = 0.005, *d* = 0.42, 95% CI [0.13, 0.69] (Fig. 6a); which suggests that vertices with low consensus convey individual-specific FC patterns. In sum, network mapping that respects, *versus* ignores, inter-individual topographical variation yielded more conservative differences attributable to age and thus may provide more precise estimates of individual-level risk using topological metrics like segregation, thereby improving clinical translation (Gratton, Kraus, et al., 2020).

## 4 Discussion

An interesting tension exists between the idea of canonical functional brain networks that are observable in individuals (Gordon, Laumann, Gilmore, et al., 2017), emerge early in neurodevelopment (Labonte et al., 2025; Sylvester et al., 2023), and exhibit correspondence across mental states and modalities (Kraus et al., 2021; Wang et al., 2015) but also have substantial inter-individual variation in spatial localization (Dworetsky et al., 2021; Hermosillo et al., 2024). This tension reflects that functional brain networks are fundamental units by which to understand brain organization across and within individuals. Network and areal boundaries tend to exhibit greater uncertainty, especially in older age in the context of cerebrovascular and anatomical differences (e.g., cortical atrophy; see network size robustness tests) (Han et al., 2018), but could also reflect accumulated experience and plasticity (Gratton & Braga, 2021). Individualized network mapping is therefore a powerful tool for characterizing neural and behavioral heterogeneity that is greater in older adulthood (Perez et al., 2025). To this point, we made several important discoveries. First, network topography in older adults was not only less like widely used group-averaged network maps, but moreover, more idiosyncratic than network topography in young adults. Age-related heterogeneity thus inflated age cohort and individual-level differences in network segregation, a widely studied measure of neurocognitive aging. Second, individualized network maps showed greater consistency within individuals over time than they do between individuals, indicating that even standard acquisition parameters (i.e., in datasets that prioritize large cohorts) capture meaningful inter-individual variation. Third, areas of high network assignment consensus are generally similar across age groups, but these areas represent only approximately half of the cortex, and some networks are more consistent across individuals (e.g., visual) than others (e.g., frontoparietal). Typical approaches assume 100% shared topography, which is why these results—consistent with prior work (Dworetsky et al., 2021; Hermosillo et al., 2024)—strikingly demonstrate not only the extent of inter-individual but also age-related variation in network topography.

Our finding that older adults are heterogeneous aligns with past work using FC weights (assuming shared topography) (Geerligs et al., 2015; Geerligs & Campbell, 2018; Hughes et al., 2025; St-Onge et al., 2023), as well as behavior, anatomy, and clinical presentation (Damoiseaux, 2017; Goh, 2011; Lowsky et al., 2014; Vogel et al., 2021; Ylikoski et al., 1999; Yu, 2024). One source of greater variability is older chronological age, as we showed across two samples comprising 176 older adults that differed, albeit modestly, in demographic composition (e.g., race, educational attainment). Even so, the correlations were moderate, which suggests that other factors contribute to heterogeneity in this period of development. Prioritizing more precise measurements of brain networks moves the field beyond age cohort comparisons, which may better leverage the rich behavioral and neural phenotyping of older adults in extant datasets. For instance, not accounting for topographical variation may explain why there is mixed evidence for prediction of individual differences in cognition, which exhibits age- and disease-related deficits (Park & Reuter-Lorenz, 2009), from intra-individual (e.g., run-to-run stability) *versus* inter-individual (e.g., similarity to high performers) features derived from FC weights (Corriveau et al., 2023; Hughes et al., 2025; Schultz & Cole, 2016a, 2016b). Future work should compare FC metrics derived from individualized (vs. group-averaged) mapping to test this possibility. In fact, we show improvements in network-function alignment even among older adults who, as a group, exhibit weaker neural activations and robust behavioral impairments related to theory of mind, a core type of social cognition (Cassidy et al., 2021; Grainger et al., 2023; Hughes et al., 2019; Moran et al., 2012). A major implication of this finding is that individualized mapping could be used to more precisely identify neural correlates underlying different symptomology in non-normative aging (e.g., episodic memory in Alzheimer’s disease versus impulsivity in frontotemporal dementia). But also, our findings reveal that segregation, a summary metric of functional brain health more broadly, is more precise at the group- and individual-level using individualized mapping. Unacknowledged heterogeneity in functional neuroanatomy limits clinical translation of fMRI-based measures (Gratton, Kraus, et al., 2020) and reduces the reliability of brain-behavior correlations (Hermosillo et al., 2024) – altogether supporting the promise of individualization to promote better understanding of older adulthood.

Another key contribution of this work was that individualized mapping yielded valid and discriminable network topography in two samples of older adults under common acquisition parameters (e.g., <20 minutes of total fMRI at 3.0T using single-echo EPI) that aim to not overburden participants who may find the scanner environment uncomfortable. This study also provided some of the first evidence, albeit in a relatively small subset of older adult participants (n = 30), that individualized maps in older adults were moderately stable and discriminable over a 2-year interval common to gold standard longitudinal studies of within-person aging (e.g. (Villeneuve et al., 2025)). This finding is important because few studies have evaluated the stability of functional network topography in any population across such a duration (see also (Lynch et al., 2024)), making it a relevant observation for aging and non-aging researchers alike.

The relatively short scan durations here precluded thorough investigation of test-retest reliability within session. Combining rest and task data to maximize data quantity generally appears to be a feasible strategy for network mapping (Du et al., 2025; Hermosillo et al., 2024). We explored this possibility in Supplementary Material analyses that propose that age group differences in topographical reliability within-session could be ameliorated by using different (*vs*. fixed) amounts of data in each group by pooling rest and movie-watching (i.e., task) fMRI data. Nonetheless, the intra-individual stability and inter-individual discriminability observed here is almost certainly lower than what could be achieved by longer acquisitions spread out over multiple sessions/days—as demonstrated in a recent report of densely-sampled older adults (Chernicky et al., 2026). Longer acquisitions can improve network mapping (i.e., topographical) reliability in multiple ways, including: (1) narrowing the reliability gap between cortex *versus* subcortex (Chernicky et al., 2026), (2) allowing for more random temporal subsampling which reduces time series autocorrelation (see Supplementary Figs. 5 and 19 in Hermosillo et al. (2024)), and (3) improving participant retention (i.e., ~40% of adolescents in the ABCD cohorts in Hermosillo et al. (2024); 28% of IU older adults were excluded from mapping due to insufficient low motion data). Multi-echo fMRI can also maximize signal and denoising quality while minimizing scan time (Lynch et al., 2021). Ultimately, these choices should be tailored to specific research goals. For instance, the current report conducted several group-level inferences about young *versus* older adults for which individualization with moderate stability may be sufficient, however, higher precision approaches would be more appropriate when group-level inference is not the aim (e.g., for neurostimulation, e.g., (Nahas et al., 2025), *de novo* network discovery (Gordon et al., 2023)). Such challenges of individualized network mapping in traditional acquisitions highlight exciting new directions for precision fMRI (Gratton & Braga, 2021; Lynch et al., 2020).

While template matching for individualized maps of functional brain networks is a common downstream tool to achieve greater fidelity to individual-specific topography, implementations vary. For example, most template matching work uses group-averaged FC templates as opposed to or in addition to the probabilistic templates used here (Betzel, 2025; Gordon, Laumann, Gilmore, et al., 2017; Lynch et al., 2024). We chose probabilistic templates because they show high consistency across a range of samples and ages (Dworetsky et al., 2021; Hermosillo et al., 2024). By contrast, FC templates may be less well-suited to age comparative studies. FC approaches involve thresholding to retain strong positive weights which can bias group comparisons because older adults have less strong positive FC than young adults (Rubinov & Sporns, 2011). Also, prior work has shown that age cohort variation in areal parcellation largely occurs at network boundaries (Han et al., 2018), which may hinder the interpretability of a winner-take-all approach (i.e., one network per voxel/vertex/cluster) because network assignments are treated with equal confidence. To highlight this problem, we visualized a single-participant’s vertex × network template similarity matrix for all cortical vertices (Fig. 1D) and one exemplar vertex (Fig. 1F). For the latter, the DMN is the clear winner, but this may not be a likely pattern for all vertices. Hermosillo et al. (2024) describes one method for decomposing this matrix to detect overlapping networks, which could be useful in future work for characterizing de-differentiated function and/or less segregated network organization in older adulthood.

Another issue is that regions with poor signal may not be a particularly good match to any network. Dilating or removing very small network clusters or not mapping vertices with poor signal—as indicators of low confidence (Lynch et al., 2024)—may mitigate this issue. For example, we noted in Supplementary Figures S12–S13 that most very small network clusters affected by dilation were found in low temporal signal-to-noise regions such as orbitofrontal cortex across all groups. That said, criterion (e.g., temporal signal-to-noise ratio) vary by acquisition, preprocessing, and age such that more work is needed to determine appropriate criterion and thresholds. Another strategy could be to use high consensus ROI sets (Figs. 5a, 6) although it is unclear if this approach is well-suited for capturing cognitive heterogeneity given that associated frontoparietal network regions are highly spatially heterogenous across individuals (Perez et al., 2025). Future work should interrogate best practices for template matching under a range of templates, acquisitions, samples, and post-processing choices. Finally, while prior-based approaches like template matching are advantageous for short acquisitions, other approaches may be better suited for different study goals or acquisitions. For example, it is possible to individualize a low dimensional number of homologous regions which may be more computationally tractable for regional graph theory metrics based on FC (Kong et al., 2019; Setton et al., 2023). While one approach is established in the aging literature and is agnostic to network labels (Setton et al., 2023), the other preserves homology of both regions and networks (Kong et al., 2019) and exhibits good correspondence with template matching (Lynch et al., 2024). Precision fMRI in developmental and clinical populations is an active area of inquiry (Chernicky et al., 2026; Demeter et al., 2025; Gratton, Kraus, et al., 2020; Gratton & Braga, 2021; Perez et al., 2025; Setton et al., 2023); the current work complements the diversity of emergent approaches and will serve as a foundation for future insights.

Beyond showing that individualized mapping is valid for studying neurocognitive aging, we also highlight how network topography itself can be a fruitful area of inquiry. Indeed, multiple investigations in young and middle aged adults, adolescents, and clinical conditions have been conducted in recent years (Chernicky et al., 2026; Demeter et al., 2025; Dworetsky et al., 2024; Hermosillo et al., 2024; Lynch et al., 2024; Nahas et al., 2025; Seitzman et al., 2019). We conducted an exploratory analysis of age differences in network size given that a recent report differentiated groups of individuals with major depressive disorder from non-clinical controls on the basis of a larger salience network (Lynch et al., 2024; Nahas et al., 2025), indicating that network size differences may be a novel risk factor for certain conditions. Taking a similar approach, we observed that network size differences between age groups appeared to be driven by trade-offs between spatially adjacent networks – termed border shifts (Kraus et al., 2021; Seitzman et al., 2019). Interestingly, the cingulo-opercular network was expanded in young adults relative to older adults, which corresponded with the near absence of high-consensus cingulo-opercular vertices in dorsal anterior cingulate cortex in older adults; an area spatially adjacent to the dorsal somatomotor network with greater surface area in older adults. Put simply, the cingulo-opercular network was displaced by the dorsal somatomotor network in older *versus* young adults. One reason these networks may be showing age differences is that a more fine-grained network topography—discoverable with higher precision approaches (Gordon et al., 2020, 2023)—exists. More work is needed to understand why differences emerge at the group- and individual-level, and the heterogeneity observed in aging is very well-suited for such investigations.

In sum, individualized mapping of functional brain networks in older adulthood is not only feasible but vital for both group and individual-level analyses of network topography and topology. Ignoring inter-individual variation in topography disproportionately mischaracterizes older adults, which we benchmarked to traditional group-averaged mappings across several metrics. That said, we also showed that individualized maps are less reliable in older than in young adults (Supplementary Material) even while evincing other positive characteristics like increased network homogeneity and moderate intra-individual stability and discriminability over time. As precision functional mapping methods proliferate, it is critical to test them on developmental and clinical populations to identify and ameliorate persistent biases that would prevent clinical translation in the groups for whom such applications are most valuable.

## Supplementary Material

Supplementary Material

## Data Availability

The IU young and older cohort data supporting the conclusions of the current work are available upon request to the first author. The IADRC cohort data supporting the conclusions of the current work could be requested via https://medicine.iu.edu/research-centers/alzheimers. The Dworetsky HCP probabilistic network templates are publicly available at www.midbatlas.io. Data files in CIFTI format supporting the visualizations of some key results are available at https://osf.io/ys28f/. The current report used the following public code repositories to generate and analyze features of individualized network maps: https://github.com/DCAN-Labs/compare_matrices_to_assign_networks (Hermosillo et al., 2024 and https://github.com/cjl2007/PFM-Depression (Lynch et al., 2024). Effect sizes and their bootstrapped 95% confidence intervals were calculated using the MATLAB Measures of Effect Size Toolbox (https://github.com/hhentschke/measures-of-effect-size-toolbox).
